# The feasibility of omitting irradiation to the contralateral lower neck in stage N1 nasopharyngeal carcinoma patients

**DOI:** 10.1186/1748-717X-8-230

**Published:** 2013-10-04

**Authors:** Weixu Hu, Guopei Zhu, Xiyin Guan, Xiaoshen Wang, Chaosu Hu

**Affiliations:** 1 Department of Radiation Oncology, Cancer Center of Fudan University, 270 Dong’an Road, Shanghai 200032, People’s Republic of China

**Keywords:** Nasopharyngeal carcinoma, Selective irradiation, Cervical lymph node metastasis

## Abstract

**Purpose:**

This study was conducted to analyze the feasibility of omitting irradiation to the contralateral lower neck in stage N1 nasopharyngeal carcinoma (NPC) patients.

**Materials and methods:**

From July 2008 to January 2012, 52 patients with stage N1 NPC were analyzed. All patients were treated with intensity-modulated radiation therapy (IMRT) and received bilateral upper neck irradiation to levels II, III and VA and ipsilateral lower neck irradiation to levels IV and VB. The contralateral lower neck irradiation was omitted.

**Results:**

The median follow-up was 29 months (range, 12–52 months). The 3-year overall survival (OS) rate, progress-free survival (PFS), local failure-free (LFS), nodal recurrence-free survival (NFS) and distant metastasis-free survival (DMFS) rates were 92.2%, 94.1%, 94.3%, 98% and 94.1%, respectively. Only one patient developed a neck recurrence in the irradiation field, while no patients experienced out-of-field nodal recurrence. Univariate analysis suggested that T classification was the only significant prognostic factor for overall survival, and age was significantly associated with PFS. Multivariate analyses indicated that age was also a predictor for overall survival. The elective neck irradiation procedure was not a significant predictor for all of the treatment results.

**Conclusion:**

Selective irradiation to bilateral levels of II, III and VA and unilateral levels of IV and VB, omitted the contralateral lower neck in a proportion of patients with N1 stage NPC was safe and practicable.

## Introduction

Nasopharyngeal carcinoma (NPC) is a highly infiltrative tumor and is prone to cervical lymph node metastasis because of the rich lymphatic network in the nasopharynx [1-3]. Traditionally, irradiation treatment of the entire cervical lymph nodal drainage region has been considered a necessity. Some studies proposed that routine irradiation to the retropharyngeal area, levels II–V and the supraclavicular lymph nodal areas were needed regardless of the nodal metastasis status [4,5].

However, based on current technology, clinical data indicate that elective neck irradiation was relatively safe and practicable. Tang et al. [6] reported that there was no statistical difference in risk for regional recurrence and distant metastasis in N0 patients with a cricoid cartilage as the inferior border of the neck irradiation field when compared with those irradiated below the cricoid cartilage. Gao et al. [7] reported that elective level II, III and VA irradiation was suitable for NPC without neck lymph node metastasis. A more recent study reported that elective irradiation to levels II, III and VA was not inferior to whole-neck irradiation for NPC patients with retropharyngeal lymph nodes metastasis only [8]. Because no consensus has been reached on the amount of irradiation required for the necks of NPC patients, it is reasonable to question the necessity of irradiation for the bilateral lower neck lymph node levels, including the inferior area of levels V and level IV and the supraclavicular regions in partial N1 patients with only unilateral upper lymph node involvement in the neck. In this study, we investigated the probability of disease recurrence in the unilateral lower neck, including levels IV and VB and supraclavicular regions in patients with positive unilateral upper cervical lymph node and explored the feasibility of reducing the irradiation range in N1 patients.

## Materials and methods

### Patient selection and pretreatment evaluation

Between July 2008 and January 2012, 52 consecutive patients were treated with intensity-modulated radiation therapy (IMRT) at the Cancer Hospital of Fudan University. The inclusion criteria for this study were: (1) histopathologically confirmed squamous cell carcinoma (SCC) of the nasopharynx; (2) T1-4N1M0 disease (according to the American Joint Committee on Cancer (AJCC) 7th staging system); (3) no previous anti-tumor therapy; (4) a Karnofsky Performance Status (KPS) ≥80; and (5) magnetic resonance imaging (MRI) pretreatment of the nasopharynx and neck.

Pretreatment evaluations included a complete medical history, physical examination, indirect or fiberoptic endoscopic examination, chest X-ray or computed tomography (CT), abdominal ultrasound or CT, MRI scans of nasopharynx and neck and complete blood counts. Bone scans were performed on patients with T3–4 disease and symptomatic patients. Patients were staged using the AJCC 7th staging system. Cervical lymph nodes were considered to be positive only if the shortest axial diameter of the jugulodigastric lymph node was ≥11 mm, the shortest axial diameter of the other lymph nodes was ≥10 mm or there was a group of three or more lymph nodes of critical size [9,10]. The lateral retropharyngeal lymph nodes (RLNs) were defined as metastatic if their shortest diameter was ≥5 mm. Any visible nodes in the median RLN were considered to be malignant [11,12], and any imaging evidence of extracapsular spread or central necrosis was also a sign of metastasis [9-12].

### Radiotherapy

All patients were treated with IMRT and immobilized in the supine position with a thermoplastic mask. CT scans were obtained from the anterior clinoid process to the hyoid bone in 3-mm slices and from the hyoid bone to 2 cm below the sternoclavicular joint in 5-mm slices. The gross tumor volume (GTV) identified on fusion MRI and CT scans included primary nasopharyngeal tumors (GTVnx) and involved lymph nodes (GTVnd). The clinical target volume (CTV) contains two parts. The CTV1 covered the entire nasopharynx, parapharyngeal space, clivus, skull base, pterygopalatine fossa, posterior half of the ethmoidal sinus, inferior sphenoid sinus, posterior one-third to one-half of the nasal cavity and maxillary sinus.

The bilateral upper neck lymph drainage region of levels II, III and VA was also included as high-risk CTV2; additionally, ipsilateral lower neck lymph node drainage areas, including levels IV and VB and the supraclavicular regions, were delineated as a low-risk CTV2. However, the contralateral lower neck and the supraclavicular lymph node drainage areas were omitted and excluded as part of the CTV. More specifically, patients without enlarged left upper cervical lymph nodes did not accept left neck lymph node irradiation of levels IV and VB. Conversely, patients without enlarged right upper cervical lymph nodes did not accept right neck lymph node irradiation of levels IV and VB.

The planning target volume (PTV) included CTV together with a margin of 3–5 mm to overcome both patient or organ motion and set-up error [13]. The prescribed doses were 66 Gy for T1-2 disease and 70.4 Gy for T3-4 disease to the PTV in the nasopharynx and 66 Gy for positive lymph nodes in 30–32 fractions. The prescribed dose for the upper neck lymph drainage region was 60 Gy, and the dose range for lower neck lymph node drainage areas was 54–60 Gy in 30–32 fractions.

The residual disease diagnosed by physical examination, including MRI, endoscopic examination and clinical examination, was treated with external-beam boost radiation using IMRT. The boost dose ranged from 6 Gy to 8 Gy in 3 to 4 fractions.

### Chemotherapy

Patients with stage II tumors received concurrent single agent chemotherapy with cisplatin 80 mg/m^2^ intravenously (IV) over 3 days every 3 weeks or 30 mg/m^2^ weekly. Patients with stage III and IV tumors received neoadjuvant chemotherapy with concurrent chemotherapy. Neoadjuvant chemotherapy mainly consisted of 2–3 cycles of TPF or TP. The TPF protocol consisted of docetaxel 75 mg/m^2^ IV and cisplatin 75 mg/m^2^ IV over 3 days and 5-fluorouracil (5-Fu) 2500 mg/m^2^ continuously IV 120 h. The TP regimen consisted of docetaxel 75 mg/m^2^ IV and cisplatin 80 mg/m^2^ IV over 3 days. The concurrent chemotherapy regimen was same as the regimen implemented in patients with stage II disease.

### Follow-up

Patients were followed up for disease status and treatment-related toxicity every 3 months in the first and second years, then every 6 months during the next 2–5 years. Each follow-up included a complete physical examination, nasopharyngoscopy or indirect nasopharyngeal speculum examination, serum biochemical profile and an abdominal ultrasound. Nasopharyngeal MRI was performed every 6 months. The chest CT and electronic epipharyngoscope were performed at least once every year. Bone scans, abdominal CT and PET-CT were performed when clinically indicated. Late toxicities were evaluated according to the toxicity criteria of the Radiation Therapy Oncology Group (RTOG) at each follow-up [14].

### Statistical analysis

All analyses were performed using the SPSS software system (version 17.0; Chicago, IL). The endpoints included overall survival (OS), local recurrence-free survival (LFS), nodal recurrence-free survival (NFS), progression-free survival (PFS) and distant metastasis-free survival (DMFS). All endpoints were defined as the interval from the treatment completion date to the date of failure or the last follow-up date. The Kaplan-Meier method was used to calculate the probabilities of OS, PFS, LFS, NFS and DFFS [15], and log-rank test performance was used to compare the differences [16]. The statistical analyses were two-sided, and P ≤0.05 was considered to be statistically significant. A multifactor Cox model was used to define the independent prognostic factors.

## Results

### Patients and treatment outcomes

A total of 52 patients with pathologically confirmed NPC were recruited in this study. The median age was 44 years (range, 26–68 years), which included 37 male and 15 female patients. The patients’ characteristics are summarized in Table 1. The study population included 29 patients with tumor stage II, 14 patients with tumor stage III and 9 patients had stage IV. Of the 52 total patients, the distribution of T stages was 6 (11.5%) stage T1, 23 (44.2%) stage T2, 14 (26.9%) stage T3 and 9 (17.3%) stage T4. All of the patients enrolled in this study were N1 stage.

**Table 1:** Patient characteristics

**Characteristics**		**Number**	**Percentile (%)**
Gender	Male	37	71.2
	Female	15	28.8
Age	Median	44	
	Range	26-68	
T stage	T1	6	11.5
	T2	23	44.2
	T3	14	26.9
	T4	9	17.3
Clinical stage	II	29	55.8
	III	14	26.9
	IV	9	17.3
Neoadjuvant chemotherapy	Yes	44	84.6
	No	8	15.4
Concurrent chemotherapy	Yes	45	86.5
	No	7	13.5
Neck boost	No	43	58.2
	EB boost	9	17.3

Fourteen patients in stage III and 8 patients in stage IV received neoadjuvant chemotherapy. Concurrent chemotherapy was implemented in 45 patients, while others refused concurrent chemotherapy for economic reasons or toxic effects such as emesis. Twenty-seven (51.9%) patients with no enlarged left upper cervical lymph node did not accept left neck lymph node irradiation of levels IV and VB. Additionally, 25 (48.1%) of the patients with no enlarged right upper cervical lymph node did not accept right neck lymph node irradiation of levels IV and VB.

### Survival and failure

**Figure 1:**
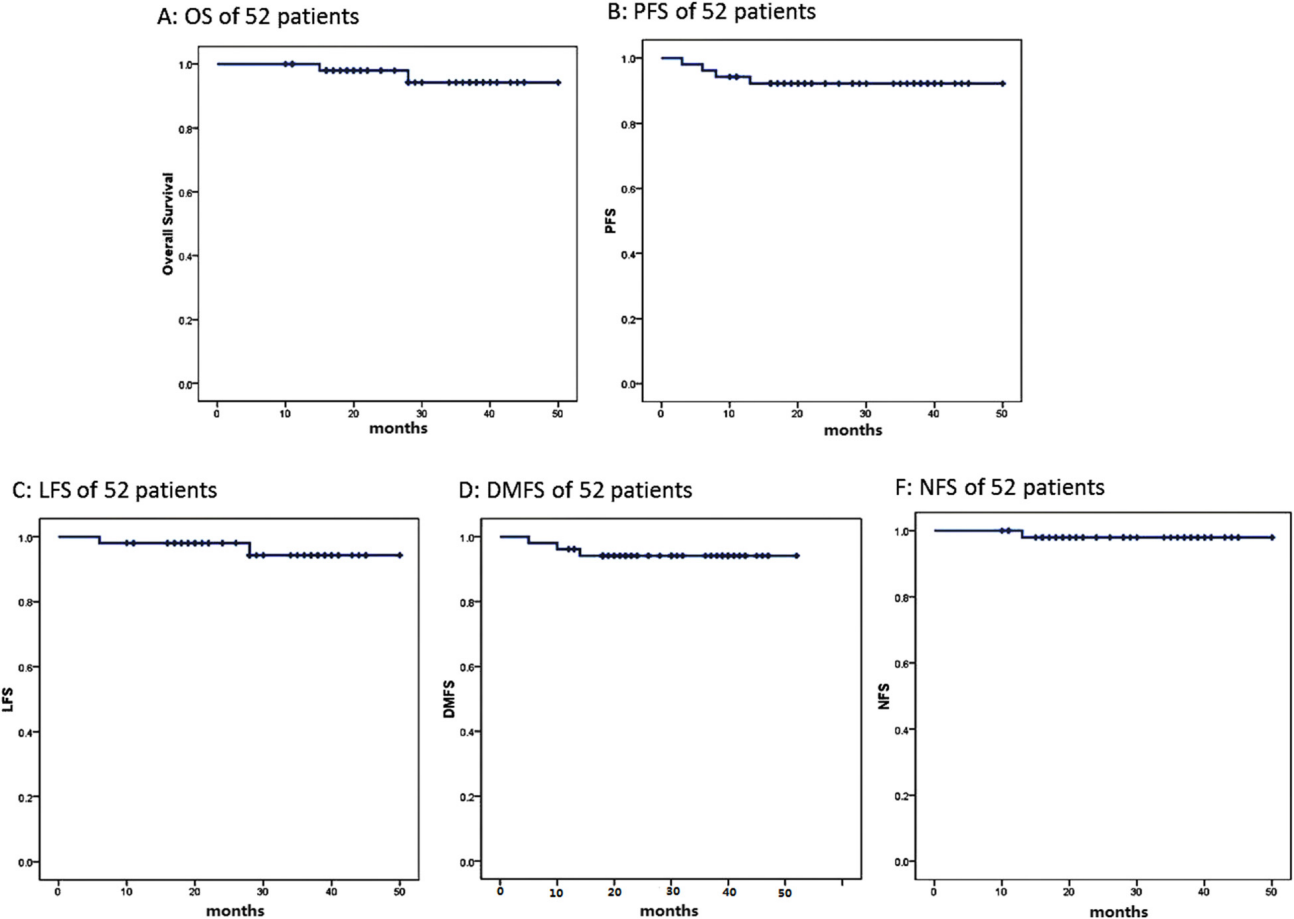
**Disease progression and overall survival in patients with stage N1 nasopharyngeal carcinoma treated with omitting irradiation to the contralateral lower neck. (A)** overall survival (OS), **(B)** progress-free survival (PFS), **(C)** local failure-free survival (LFS), **(D)** distant metastasis-free survival(DMFS), and **(F)** nodal recurrence-free survival (NFS).

The median follow-up time for the entire group was 29 months (range, 12–52 months). Table 2 summarizes the failure pattern. Among all of the patients, 3 patients who had residual nasopharyngeal disease and 9 patients who had residual cervical lymph node disease received an external beam boost. At the time of this analysis, only 1 patient experienced nodal recurrence in level II, 1 patient developed primary area disease recurrence and distant metastasis occurred in 3 patients. Additionally, none of the patients experienced an out-of-radiation-field nodal disease recurrence. Three patients died, and the major failure pattern was distant metastasis: 1 patient developed nasopharyngeal disease recurrence and was treated with external beam boost, though the patient eventually died from distant lung metastasis after salvage chemotherapy; one patient experienced hepatic metastasis; another patient suffered in-field neck disease recurrence and received neck lymphadenectomy. The 3-year OS rate of the whole cohort was 92.2%, and the 4-year PFS, LFS, NFS and DMFS were 94.1%, 94.3%, 98% and 94.1%, respectively (Figure 1).

**Table 2:** Failure patterns and sites of distant metastasis

**Failure**		**Frequency**	**Percentile (%)**
Local recurrence		1	1.9
Local & nodal recurrence		0	0
Nodal recurrence	In-field	1	1.9
	Out-of-field	0	0
Local recurrence & distant metastasis		1	1.9
Distant metastasis		3	5.8
	Lung	1	1.9
	Liver	2	3.8
Deaths		3	5.8

### Late toxicities

With the IMRT technique, the late toxicities observed were generally mild or moderate; the most common acute toxicities were grade 1–2 mucositis, whereas 7 (13.5%) patients had grade 1–2 xerostomia, 9 (17.3%) had subcutaneous fibrosis and 6 (11.5%) had tinnitus. None of the patients suffered from cranial neuropathy, trismus or brain damage, and no grade III-IV late toxicities were observed.

### Univariate and multivariate analyses

The data used for univariate and multivariate analyses included the patients’ age, sex, T staging, energy beam boost and chemotherapy, and possible prognostic factors are presented in Table 3. Univariate analyses revealed that that T classification was the only significant prognostic factor for overall survival, and age significantly influenced the PFS. Additionally, there was a tendency toward decreased local control with increased T staging, although the P value did not reach statistical significance. This result may be due to the small sample size in this series. The age (>44 years) was also a predictor for OS in the multivariate analysis (P=0.045, hazard ratio [HR] 1.176, 95% confidence interval [CI] 1.003-1.378). However, the local or nodal recurrence was not affected by any other prognosticator that we studied, such as age, sex, T classification or chemotherapy.

**Table 3:** Univariate analysis of prognostic factors

	**3-y OS**		**3-y LFS**		**3-y NFS**		**3-y DFFS**		**3-y PFS**	
	**%**	**P**	**%**	**P**	**%**	**P**	**%**	**P**	**%**	**P**
Gender		0.777		0.379		0.584		0.814		0.908
M	92.1		92.2		97.2		94.5		91.8	
F	92.3		100		100		93.3		93.3	
Age		0.069		0.125		0.327		0.079		0.040
≧44	83.6		87.4		96.0		88.3		84.4	
<44	100		100		100		100		100	
T stage		0.037		0.078		0.268		0.429		0.199
T1-T2	100		100		100		96.6		96.6	
T3-T4	80.8		85.0		95.5		91.1		86.7	
Chemotherapy		0.471		0.531		0.709		0.497		0.428
Yes	90.8		93.1		97.7		93.2		91.0	
No	100		100		100		100		100	
Boost		0.711		0.366		0.079		0.330		0.969
Yes	83.3		83.3		91.7		100		91.7	
No	94.6		97.5		100		92.3		92.5	

## Discussion

Radiation therapy is a mainstay in the treatment of NPC. Irradiation from the bilateral upper cervical lymph node areas down to the supraclavicular fossa has long been the standard treatment model for radiation therapy of NPC [17-19] because of the abundant supply of lymphatic networks in the nasopharynx. This methodology is based on reports by Nq WT et al. and Tang L et al. who had confirmed cervical lymph adenopathy in more than 85% patients with NPC, while approximately 50% patients demonstrated bilateral cervical lymph node metastasis [6,20,21] in an occult cervical lymph node metastatic model. The limitations of previous studies include the following: first, due to the poor imaging techniques, most patients were staged by clinical palpation; and second, outdated radiation techniques led to an inhomogeneous dose in the target, which may cause poor regional control.

Recently, studies have reported radiation therapy with local and regional control and with OS rates of approximately 85%, 90% and 80% with or without chemotherapy [22-25]. Several articles subsequently addressed the probability that skip metastasis in the cervical nodal areas was extremely low, with ranges between 0.2% and 7.9%; moreover, cervical lymph node metastases in NPC always follow an orderly spreading pattern [6,20,26-28]. Additionally, the most common regions for cervical lymph node metastasis were the RLNs and level II at frequencies of 69% and 70%, respectively. However, only 11% of patients developed cervical lymph node metastasis in level IV [29]. Subsequently, Su SF et al. [30] reported that in NPC patients with N0 disease, there was no regional disease recurrence even if radiation was applied to the primary tumor and upper neck nodal areas only, while the 5-year regional control, local control and OS rates were 95.6%, 93.4% and 89.8%. Moreover, Yunsheng Gao et al. [7] and Xiaomin Ou et al. [8] demonstrated that elective neck irradiation to levels II, III and VA was not only suitable for patients with N0 disease but also appropriate for patients with only RLN metastasis; with this treatment, the out-of-field recurrence rates were 0.2% and 0.84%, respectively. Additionally, the authors described high local control rates of 88.6% and 81.4% respectively, while the OS rates were 84.2% and 93.6%.

In consideration of the previous results, it is reasonable to challenge the necessity of whole bilateral cervical lymph node area irradiation. This is the first time our research focused on the cervical irradiation range in N1 patients with unilateral cervical lymph node metastasis. The assumption underlying the design of our trial was that irradiation only to the bilateral upper cervical nodal areas and unilateral lower neck without prophylactic treatment to the other side of the lower neck in unilateral cervical metastasis is feasible. Our analysis showed excellent local and regional tumor control for N1 NPC patients with only unilateral upper cervical lymph node metastasis who were treated with radiotherapy to the bilateral upper cervical nodal areas (levels II, III and VA) and the unilateral lower neck, where ipsilateral upper neck lymph node metastasis existed (levels IV and VB down to supraclavicular fossa) and for patients with primary disease. The 3-year overall survival and local control rates were 92.2% and 94.3%. More importantly, only one patient experienced nodal disease recurrence in the irradiation field, and no patients experienced out-of-field recurrence. Furthermore, the 3-year NFS rate was 98%. Our results were similar to Gao et al. [7] and Chen et al. [31], who also reported low nodal recurrence rates of 3.4% and 3.3%. Univariate analyses suggest that T classification was the only significant prognostic factor for predicting overall survival and that age was a predictor of PFS. Moreover, we observed a tendency for decreased local tumor control with increasing T classification; however, the P value did not indicate statistical significance, which we attributed to the small sample size. Multivariate analysis indicated that elective neck irradiation was not a significant predictor for local or nodal control. However old age was an independent predictor for OS; this result corresponded with previous reports [8,32]. Here, we concluded that elective neck irradiation was not inferior to irradiation of the entire bilateral cervical lymph node area.

As a foundation of the IMRT technique, an accurate cervical lymph node metastasis staging for NPC is crucial. Previously, the traditional CT scan was a common method for NPC staging and for designing therapeutic strategies. Today, however, the multi-slice spiral CT scan, which has excellent resolution, and MRI have become the mainstream methods and have replaced the traditional CT scan. MRI, with its higher sensitivity, is superior to CT scans for detecting tumor extensions, soft-tissues and critical structures [33,34]. Here, we combined contrast-enhanced CT and MRI, and we estimated primary tumor and cervical lymph node metastasis accurately, which provides more potential for implementing precise radiotherapy. In addition to the new techniques for oncologic imaging, IMRT plays a vital role in tumor control. Lee N et al. confirmed that through delivery of high doses up to the target volume without affecting normal structures, IMRT may provide better tumor control [35]. Additionally, Ou et al. reported that IMRT displayed the capacity to improve regional control [8]. For NPC, the doses for potential risk sites, such as subclinical cervical areas, were vital for regional control; the consensus opinion is that a dose of 50–60 Gy is necessary [36]. In the Ou et al. study [8], the dose for the upper neck was the independent prognostic factor of NFS in patients with only RLN metastasis. The authors regarded upper-neck lymph areas as potential risk sites and treated them with a relatively higher dose [8]. A possible explanation for these observations was that once the unilateral upper neck developed lymph node metastases, the risk of regional recurrence and metastasis of the contralateral upper neck area and ipsilateral lower neck area was higher; therefore, an adequate dose for these areas was crucial. In our study, we directed a relatively higher dose (60 Gy) to bilateral upper cervical nodal areas and a moderate dose (54 Gy) to the unilateral lower neck with lymph node metastasis exists in the ipsilateral upper . The combination of CT and MRI scans was useful in planning treatments and allowed the accurate image definition of tumor tissues. The new IMRT technique provided a better dose distribution, which may contribute to regional tumor control. Based on the above results for remarkable nodal control and no out-of-field disease recurrence, irradiation to the bilateral upper cervical nodal areas and unilateral lower neck without prophylactic treatment to the contralateral lower neck in patients with unilateral cervical metastasis was appropriate.

The aims of IMRT were to optimize the dose for the target and to reduce the dose received by normal tissue. The consensus accepted doses for these potential risk sites ranged from 50 Gy to 60 Gy [4,23]. However, the irradiation field of the lower neck usually encompasses the apex of the double-lung, part of trachea, the thyroid gland, muscle and skin. Although the tolerance doses for the trachea, thyroid gland, muscle and skin usually exceed the doses for potential risk sites, the apex of the double-lung usually has a dose that exceeds the tolerance and could be adversely affected. Even in muscle, which was considered to tolerate a higher radiation dose, higher dosages have been reported to be related to peripheral neuropathy and, subsequently, decreased quality of life after irradiation treatment [37]. Although our study lacked a control group, our data demonstrated that the rates of certain late toxicities, including skin dystrophy, subcutaneous fibrosis, cranial neuropathy and trismus, were low. However, because of the short follow-up time, various late toxicities, such as temporal lobe necrosis, hypothyroidism, brachial plexopathy and hypo-pituitarism, could not be observed sufficiently.

In conclusion, in terms of excellent regional control and the lack of out-of-field disease recurrence, our prospective study demonstrated that elective irradiation to bilateral levels of II, III and VA and unilateral levels of IV and VB, omitted the contralateral lower neck in a proportion of patients with N1 stage tumors was safe and practicable.

## Consent

Written informed consent was obtained from the patient for the publication of this report and any accompanying images.

## Competing interests

The authors declare that they have no competing interests.

## Authors’ contributions

WH contributed in the collection and analysis of data and drafting the manuscript; XG, KZ and CH helped with the initial design of the study and provided critical revision of the manuscript; GZ provided the conception of this study and the final approval of the version to be published. All authors have read and approved the final manuscript.
